# Biological Activity, Lipophilicity and Cytotoxicity of Novel 3-Acetyl-2,5-disubstituted-1,3,4-oxadiazolines

**DOI:** 10.3390/ijms222413669

**Published:** 2021-12-20

**Authors:** Kinga Paruch, Anna Biernasiuk, Anna Berecka-Rycerz, Anna Hordyjewska, Łukasz Popiołek

**Affiliations:** 1 Chair and Department of Organic Chemistry, Faculty of Pharmacy, Medical University of Lublin, 4A Chodźki Street, 20-093 Lublin, Poland; lukasz.popiolek@umlub.pl; 2 Chair and Department of Pharmaceutical Microbiology, Faculty of Pharmacy, Medical University of Lublin, 1 Chodźki Street, 20-093 Lublin, Poland; anna.biernasiuk@umlub.pl; 3 Chair and Department of Medicinal Chemistry, Faculty of Pharmacy, Medical University of Lublin, 4 Jaczewskiego Street, 20-093 Lublin, Poland; anna.berecka@umlub.pl; 4 Chair and Department of Medicinal Chemistry, Faculty of Medical Sciences, Medical University of Lublin, 4A Chodźki Street, 20-093 Lublin, Poland; anna.hordyjewska@umlub.pl

**Keywords:** *N*-acetyl-1,3,4-oxadiazoline derivatives, antimicrobial activity, lipophilicity, cytotoxicity, acylhydrazone

## Abstract

Antibiotic resistance is now a global problem, and the lack of effective antimicrobial agents for the treatment of diseases caused by resistant microbes is increasing. The 3-acetyl-2,5-disubstituted-1,3,4-oxadiazolines presented in this article may provide a good starting point for the development of potential new effective antimicrobial agents useful in the treatment of bacterial and fungal infections. Particular attention is drawn to the 1,3,4-oxadiazole derivative marked with the number **29** with 5-nitrofuran-2-yl substituent in its chemical structure. This substance showed a strong bactericidal effect, especially against *Staphylococcus* spp., and no cytotoxicity to the L929 normal cell line.

## 1. Introduction

The overuse and misuse of antibiotics has caused one of the most serious global threats—antimicrobial resistance [1,2]. Antimicrobial resistance is among the principal factors involved in the persistence of chronic infections [3]. This problem becomes more serious for immunocompromised patients and those who are often disposed to opportunistic infections [4]. The most significant fact associated with this phenomenon is the reduction of the number of effective antimicrobial drugs, which has led to an increase in therapeutic problems, complications and an increase in mortality [5,6,7]. Moreover, there is a huge disproportion between the frequency of the introduction of new antibiotics into treatment and the rate of the development of bacterial and fungal resistance. Therefore, in order to maintain the effectiveness of the treatment of bacterial infections as long as possible, it is necessary to take preventive measures, but to also search for new forms of therapy. If antibiotics with reduced and documented action become ineffective, new substances with high effectiveness that are safe for patients should be discovered [5].

Bearing in mind the above-mentioned problems, we conducted a literature review that showed that 3-acetyl-1,3,4-oxadiazoline derivatives constitute the class of compounds with high biological potential [8]. A simple method of the synthesis and a broad spectrum of activity make them an intensively studied group [9]. Our literature review confirmed their effectiveness. The mechanism of antimicrobial action is likely based on the presence of the -N=CO group in their chemical structure and its influence on the transcription of genes involved in biofilm formation, especially against *Staphylococcus aureus* [10]. According to the literature, antibacterial [11,12,13,14,15,16], antifungal [17,18,19,20], anticancer [21,22,23], antimycobacterial [24,25,26,27] and antiprotozoal [28,29] effects of 1,3,4-oxadiazoles have been documented. Currently, there are no registered medicines with an acetyl substituent in the 1,3,4-oxadiazole ring, but there are medicines with an 1,3,4-oxadiazole moiety, such as Furamizole, Nesapidil, Zibotentan, Raltegravir or Tiodazosin [8]. The introduction of an acetyl group in the third position of the 1,3,4-oxadiazole ring seems to be an interesting solution, especially with regard to the fact that many studies showed that it can induce antimicrobial activity. This relationship can be observed in the article by Chawla et al. [14], where the authors compare the antimicrobial activity of 1,3,4-oxadiazoles in relation to *N*-acetyl-1,3,4-oxadiazoles. Compounds with an acetyl group showed significantly greater antimicrobial activity against all tested strains, and two of the synthesized compounds were more active than ciprofloxacin, which was used as the reference substance [14]. Taking into account all these aspects, we decided to synthesize a group of compounds with the 3-acetyl-1,3,4-oxadiazole system and evaluate their antimicrobial activity and cytotoxicity, and we decided to determine their lipophilicity, especially due to the fact that it is one of the most important parameters used in the prediction of the biological activity of a given substance, as well as its toxicity [30,31].

## 2. Results

### 2.1. Chemistry

**Scheme 1:**
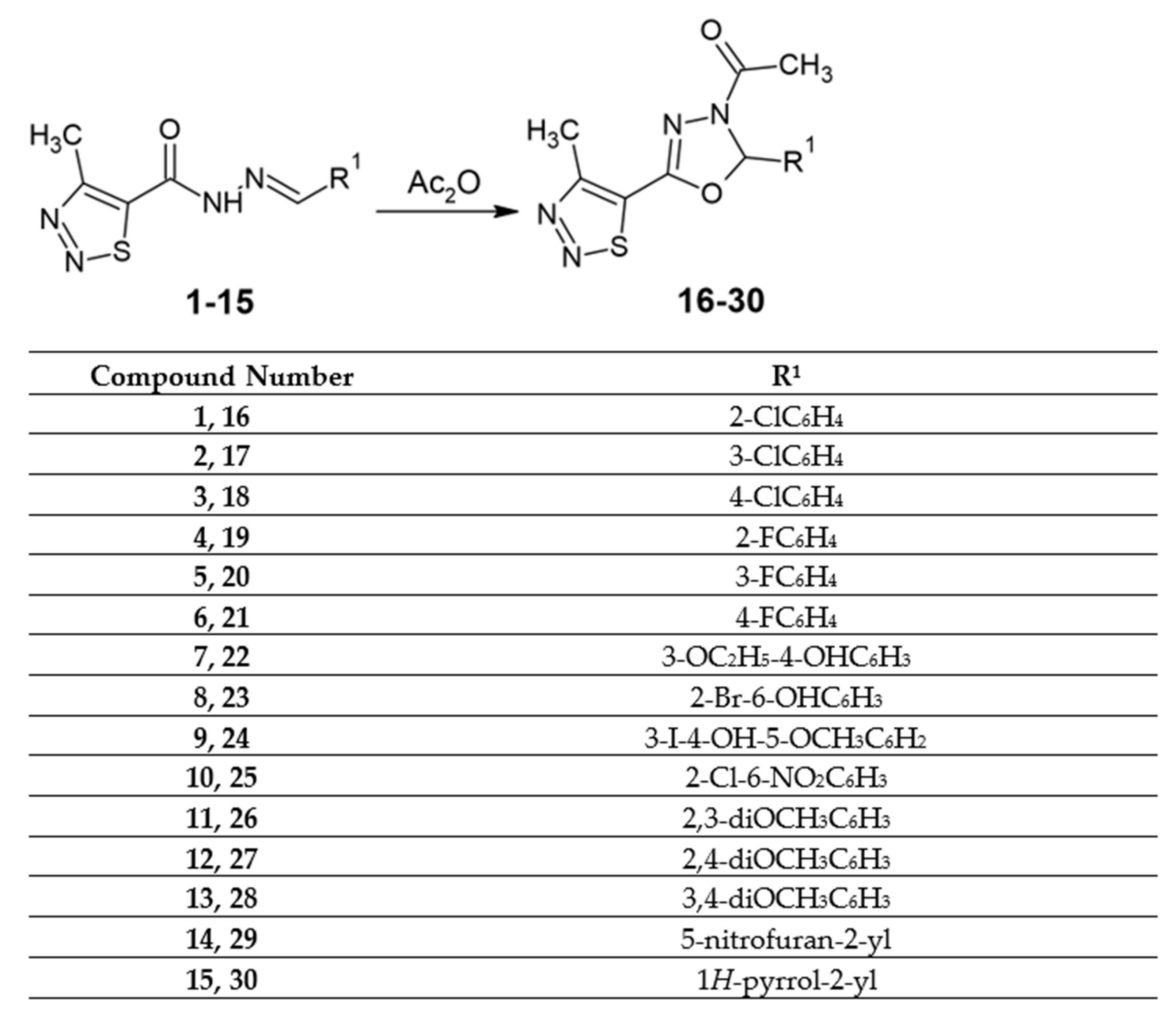
Synthesis scheme of novel derivatives of 3-acetyl-2,5-disubstituted-1,3,4-oxadiazoline.

For the purpose of this study, we synthesized new 3-acetyl-2,5-disubstituted-1,3,4-oxadiazoline derivatives in the cyclization reaction with pure acetic anhydride of the previously described acylhydrazones [32] (Scheme 1). Novel 1,3,4-oxadiazole derivatives were obtained with 48–70% yields. All synthesized compounds are solids and can be dissolved in DMSO at room temperature.

In order to confirm the chemical structure of the obtained compounds, elemental analysis as well as ^1^H NMR, ^13^C NMR and FT-IR spectra analyses were performed.

On the basis of the characteristic signals presented in the ^1^H NMR, ^13^C NMR and FT-IR spectra, we determined the positive course of the cyclization reaction and proper synthesis of new 3-acetyl-2,5-disubstituted-1,3,4-oxadiazoline derivatives.

In the ^1^H NMR spectra, we observed a singlet signal for the acetyl substituent of the methyl group at δ 2.87–2.99 ppm, and for the CH group of 1,3,4-oxadiazoline, in the range of δ 7.18–8.85 ppm. In the ^13^C NMR spectra, we confirmed the presence of the carbon atom of CH group and the carbon atom of the 1,3,4-oxadiazole ring at δ 85.01–113.12 and 151.54–160.26 ppm, respectively. Additionally, we observed characteristic signals of the following fragments, such as C=O, C=N and C-OC in the FT-IR spectra.

### 2.2. Microbiology

The obtained 3-acetyl-2,5-disubstituted-1,3,4-oxadiazole derivatives were subjected to a series of microbiological tests against a panel of Gram-positive bacteria, Gram-negative bacteria and fungi from *Candida* spp. A panel of reference strains of microorganisms also included some resistant staphylococci—methicillin-resistant *Staphylococcus aureus*—MRSA ATCC 43300.

The obtained results are presented in Table 1 and they indicate that newly synthesized compounds **16**–**30** exhibited some antimicrobial activity. Among them, compounds **23** and **29** showed the widest spectrum of activity against all microorganisms, except rods from *Pseudomonas aeruginosa* ATCC 9027. Gram-positive bacteria were more sensitive to these substances than Gram-negative bacteria and fungi. The substance **29** indicated the highest antibacterial activity with minimal inhibitory concentrations (MIC), ranging from 3.91 to 250 µg/mL, and minimal bactericidal concentrations (MBC) from 15.62 to 1000 µg/mL with a bactericidal effect (MBC/MIC = 1–4) toward all reference Gram-positive bacteria. This compound had strong or very strong activity against staphylococci or bacilli. Compound **29** showed strong activity with MIC = 15.62 µg/mL and MBC = 31.25 µg/mL toward *S. aureus* ATCC 43300 with bactericidal effect. *Staphylococcus epidermidis* ATCC 12228 was the most sensitive to this derivative (MIC = 3.91 µg/mL and MBC = 15.62 µg/mL). The activity toward rods from *Enterobacterales* family belonging to Gram-negative bacteria was slightly weaker (MIC = 62.5–500 µg/mL and MBC = 125 to >2000 µg/mL) with bactericidal or bacteriostatic effect. The activity of compound **29** against most of the strains was comparable to the reference substance—nitrofurantoin. Additionally, in Supplementary Materials, we have presented antimicrobial activity of compound **29** as figures (Figures S1 and S2).

In the case of compound **23**, activity was good against *Micrococcus luteus* ATCC 10240 and *Bordetella bronchiseptica* ATCC 4617 (MIC = 62.5–125 µg/mL, MBC = 1000–2000 µg/mL and MBC/MIC = 8–16) and moderate or mild toward other bacteria (MIC = 500–1000 µg/mL and MBC = 1000 or ≥2000 µg/mL) with bactericidal or bacteriostatic effects. The remaining compounds **19**, **21**, **22**, **24**, and **25** showed some antimicrobial effect only against certain Gram-positive bacteria.

On the basis of obtained data, it was shown that some substances also had antifungal activity against yeasts belonging to reference *Candida* spp. In the case of the compounds **23**, **24** and **29**, growth of all fungal strains was inhibited at MIC = 62.5–1000 µg/mL. In turn, minimal fungicidal concentrations (MFC) ranged from 62.5 µg/mL to >2000 µg/mL. The activity of substances **23** and **29** was the highest, with good fungicidal effect toward *C. albicans* ATCC 2091 and *C. albicans* ATCC 10231. Additionally, mild activity was displayed by the compound **25** against *C. parapsilosis* ATCC 22019.

Moreover, remaining newly synthesized compounds, namely **16**–**18**, **20**, **26**–**28** and **30**, had no activity against all reference microorganisms.

### 2.3. Cytotoxicity Studies

The most active compounds were tested for cytotoxicity. The 24 and 48 h culture incubation of L929 cells with 3-acetyl-2,5-disubstituted-1,3,4-oxadiazolines (**24**, **25**, **29**), showed that compound **25** at a concentration of 100 µM (24 h) and 200 µM (48 h) was the most toxic for this line. The remaining compounds did not significantly affect cell cytotoxicity, and sometimes led to an increase in viability—the compound **24** at a concentration of 12 and 6 µM after 24 h and after 48 h at 6 µM, and **29**—at a concentration of 50 µM (Table 2). The 24 and 48 h culture incubation of A549 and HepG2 cells with tested substances showed that obtained 1,3,4-oxadiazoline derivatives significantly stimulated cell viability. In both systems tested, values above 100% cell viability were mostly obtained (Table 3 and Table 4).

### 2.4. Lipophilicity

Chromatographic methods are good for determining experimental lipophilicity. Due to their speed and repeatability, they allow to determine the lipophilicity of a wide range of newly synthesized compounds. This study was based on a standardization procedure with the use of six reference substances in the lipophilicity range of 0.46 to 3.8 [33]. As a result, it gave a strong correlation of the log P value with R_M0_ in solvent systems containing various organic modifiers, i.e., acetone, acetonitrile, 1,4-dioxane and methanol (Table 5), and finally, respective calibration curves for further lipophilicity study were obtained:

(1) acetone: log P_EXP_ = 0.8945 × R_M0_ + 0.1651; *r*^2^ = 0.9241;
(2) acetonitrile: log P_EXP_ = 2.2154 × R_M0_ − 1.6825; *r*^2^ = 0.9459;
(3) 1,4-dioxane: log P_EXP_ = 0.9387 × R_M0_ + 0.6354; *r*^2^ = 0.9653;
(4) methanol: log P_EXP_ = 0.9344 × R_M0_ + 0.2411; *r*^2^ = 0.9442.

The correlation coefficients (*r*^2^) for presented equations for the reference compounds were above 0.92 for all organic modifiers used, i.e., acetone, acetonitrile, 1,4-dioxane and methanol. Similarly, the correlations between the R_F_ and R_M0_ for all newly tested 1,3,4-oxadiazoline derivatives were also sufficiently high (*r*^2^ > 0.91) for all chromatographic systems. In addition, better correlations, i.e., *r*^2^ > 0.96, were obtained for almost all derivatives, providing accuracy for further lipophilicity determination (Table 6).

On the basis of the above-presented calibration equations and respective R_M0_ values, experimental lipophilicity (log P_EXP_) of fourteen 1,3,4-oxadiazoline derivatives (**16**–**22**, **24**–**30**) was calculated. The final results of our lipophilicity experiments are shown in Table 7. For all fourteen compounds, the obtained log P_EXP_ values were close to the R_M0_ values in the case of solvent systems, which contained acetone or methanol as an organic modifier. As a result, the obtained values of lipophilicity can be considered as reliable. For the solvent systems with acetone or methanol, the highest values of lipophilicity were obtained for the compounds **17**, **18** and **24**, and among them for the compound **24** containing an iodine atom. In addition, for the compounds **17** and **18**, the position of the chlorine atom (*meta-* or *para-*) in the phenyl ring did not visibly affect lipophilicity. However, for the *ortho-* substituted chlorine isomer (the compound **16**), the lowest lipophilicity was obtained, not only in acetone and methanol, but in all solvent systems used. For the fluorine substituted compounds (**19**, **20**, **21**), lower lipophilicity values were obtained versus for the chlorine derivatives, with no difference for the individual *ortho-*, *meta-* and *para-* isomers. Finally, the additional substitution of the chlorine derivative with the nitro group increased the lipophilicity, which was observed for the pair of compounds **16** and **25**. In general, it was observed that the antibacterial activity of synthesized compounds was not dependent on their lipophilicity.

## 3. Material and Methods

### 3.1. Chemistry

All reagents used in the experiments in this research were purchased from Sigma-Aldrich (Munich, Germany) and Merck Co. (Darmstadt, Germany) and used without further purification. They had class of purity declared by the manufacturer. The purity of the obtained compounds was assessed by means of thin layer chromatography (TLC) on plates covered with silica gel (aluminum oxide 60 F-254) delivered by Merck Co. Chloroform–ethanol mixture in the 10:1 (*v*/*v*) ratio was used as the mobile phase. The spots were detected by irradiation with UV light at a wavelength of λ = 254 nm. The FT-IR spectra were recorded on a Nicolet 6700 spectrometer (Thermo Scientific, Madison, WI, USA); in cm^−1^. The ^1^H and ^13^C NMR spectra were recorded on the Bruker Avance 300 and 600 apparatus (Bruker BioSpin GmbH, Ettlingen, Germany). The melting points of the obtained compounds were determined with a Fisher–Johns apparatus (Fisher Scientific, Waltham, MA, USA) and were presented without any correction. The compounds were dissolved in dimethylsulfoxide (DMSO-*d_6_*) for the analysis. Tetramethylsilane (TMS) was used as an internal standard. Chemical shift values are given in ppm. The elemental analysis was determined by a Perkin Elmer 2400 series II CHNS/O analyzer (Waltham, MA, USA), and the results were within ± 0.4% of the theoretical values.

#### Synthesis of 3-Acetyl-2,5-disubstituted-1,3,4-oxadiazolines

Previously obtained, 0.001 mole of appropriate acylhydrazone (**1**–**15**) [32] was dissolved in 3 mL of neat acetic anhydride and heated under reflux for 3 h. Subsequently, the acetic anhydride was removed under reduced pressure. Crushed ice was added to the liquid remaining in the flask and was shaken vigorously for 15 min. The resulting mixture was allowed to stand at room temperature for 24 h. After that, the precipitate formed was filtered under pressure and crystallized from ethanol. The crude solid was transferred to a round bottom flask and heated under reflux until it dissolved in the appropriate amount of ethanol (96%). Then, undissolved impurities were filtered off, and the filtrate was cooled to room temperature to precipitate crystals which were then filtered off under reduced pressure. The product was assessed for purity by TLC chromatography.

Detailed physico-chemical properties of new derivatives of 3-acetyl-2,5-disubstituted-1,3,4-oxadiazoline derivatives (**16**–**30**)

1-[2-(2-chlorophenyl)-5-(4-methyl-1,2,3-thiadiazol-5-yl)-1,3,4-oxadiazol-3(2*H*)-yl]ethan-1-one (**16**)

White powder, Yield: 54%, M.p.: 100 °C; IR: 3031 (CH_arom_), 2970 (CH_aliph_), 1644 (C=O), 1599 (C=N), 1277, 1046 (C-OC); ^1^H NMR (600 MHz, DMSO-*d_6_*): 2.30 (s, 3H, CH_3_), 2.98 (s, 3H, CH_3_), 7.52–7.54 (m, 1H, ArH), 7.56–7.60 (m, 1H, ArH), 8.13–8.14 (m, 1H, ArH), 8.63 (s, 1H, CH_oxadiazole_); ^13^C NMR (75 MHz, DMSO-*d_6_*): 11.56 (CH_3_), 14.95 (CH_3_), 90.91 (CH_oxadiazole_), 128.47, 130.72, 132.25, 132.56, 137.38, 141.88, 148.28 (7C_ar_), 155.13 (C_oxadiazole_), 158.50 (C_ar_), 163.36 (C=O); Anal. calc. for C_13_H_11_ClN_4_O_2_S (322.77) (%): C 48.37; H 3.44; N 17.36. Found: C 49.25; H 3.31; N 17.50.

1-[2-(3-chlorophenyl)-5-(4-methyl-1,2,3-thiadiazol-5-yl)-1,3,4-oxadiazol-3(2*H*)-yl]ethan-1-one (**17**)

White powder, Yield: 62%, M.p.: 196 °C; IR: 3069 (CH_arom_), 2970 (CH_aliph_), 1636 (C=O), 1577 (C=N), 1216, 1036 (C-OC); ^1^H NMR (600 MHz, DMSO-*d_6_*): 2.50 (s, 3H, CH_3_), 2.98 (s, 3H, CH_3_), 7.57–7.59 (m, 2H, ArH), 7.83–7.85 (m, 2H, ArH), 8.22 (s, 1H, CH_oxadiazole_); ^13^C NMR (150 MHz, DMSO-*d_6_*): 11.66 (CH_3_), 14.97 (CH_3_), 91.72 (CH_oxadiazole_), 126.07, 127.33, 130.66, 131.41, 133.95, 137.44, 138.08 (7C_ar_), 155.24 (C_oxadiazole_), 158.87 (C_ar_), 163.37 (C=O); Anal. calc. for C_13_H_11_ClN_4_O_2_S (322.77) (%): C 48.37; H 3.44; N 17.36. Found: C 48.62; H 3.41; N 18.10.

1-[2-(4-chlorophenyl)-5-(4-methyl-1,2,3-thiadiazol-5-yl)-1,3,4-oxadiazol-3(2*H*)-yl]ethan-1-one (**18**)

White powder, Yield: 48%, M.p.: 94 °C; IR: 3034 (CH_arom_), 2970 (CH_aliph_), 1667 (C=O), 1520 (C=N), 1206, 1090 (C-OC); ^1^H NMR (600 MHz, DMSO-*d_6_*): 2.28 (s, 3H, CH_3_), 2.92 (s, 3H, CH_2_), 7.22–7.25 (d, 1H, ArH, *J* = 18 Hz), 7.52–7.55 (m, 2H, ArH), 7.57 (s, 1H, CH_oxadiazole_), 7.56–7.58 (m, 1H, ArH); ^13^C NMR (75 MHz, DMSO-*d_6_*): 14.33 (CH_3_), 14.98 (CH_3_), 92.61 (CH_oxadiazole_), 129.42, 135.17, 135.32, 148.49, 155.07 (7C_ar_), 155.24 (C_oxadiazole_), 159.63 (C_ar_), 163.35 (C=O); Anal. calc. for C_13_H_11_ClN_4_O_2_S (322.77) (%): C 48.37; H 3.44; N 17.36. Found: C 48.92; H 3.51; N 18.21.

1-[2-(2-fluorophenyl)-5-(4-methyl-1,2,3-thiadiazol-5-yl)-1,3,4-oxadiazol-3(2*H*)-yl]ethan-1-one (**19**)

White powder, Yield: 67%, M.p.: 134 °C; IR: 3008 (CH_arom_), 2938 (CH_aliph_), 1636 (C=O), 1517 (C=N), 1205, 1092 (C-OC); ^1^H NMR (600 MHz, DMSO-*d_6_*): 2.22 (s, 3H, CH_3_), 2.92 (s, 3H, CH_3_), 7.30–7.36 (m, 2H, ArH), 7.54–7.59 (m, 2H, ArH), 8.44 (s, 1H, CH_oxadiazole_); ^13^C NMR (75 MHz, DMSO-*d_6_*): 11.54 (CH_3_), 14.95 (CH_3_), 89.02 (CH_oxadiazole_), 116.51, 122.57, 125.41, 130.07, 137.37, 141.89, 154.91 (7C_ar_), 158.57 (C_oxadiazole_), 160.23 (C_ar_), 163.34 (C=O); Anal. calc. for C_13_H_11_FN_4_O_2_S (306.32) (%): C 50.97; H 3.62; N 18.29. Found: C 49.85; H 3.31; N 19.50.

1-[2-(3-fluorophenyl)-5-(4-methyl-1,2,3-thiadiazol-5-yl)-1,3,4-oxadiazol-3(2*H*)-yl]ethan-1-one (**20**)

White powder, Yield: 78%, M.p.: 130 °C; IR: 3062 (CH_arom_), 2929 (CH_aliph_), 1672 (C=O), 1522 (C=N), 1215, 1061 (C-OC); ^1^H NMR (600 MHz, DMSO-*d_6_*): 2.24 (s, 3H, CH_3_), 2.86 (s, 3H, CH_3_), 7.22 (s, 1H, CH_oxadiazole_), 7.31–7.35 (m, 1H, ArH), 7.49–7.43 (m, 2H, ArH), 7.51–7.55 (m, 1H, ArH); ^13^C NMR (75 MHz, DMSO-*d_6_*): 11.67 (CH_3_), 15.00 (CH_3_), 91.73 (CH_oxadiazole_), 114.49, 115.79, 123.51, 131.53, 137.44, 138.83, 155.19 (7C_ar_), 158.83 (C_oxadiazole_), 161.01 (C_ar_), 163.37 (C=O); Anal. calc. for C_13_H_11_FN_4_O_2_S (306.32) (%): C 50.97; H 3.62; N 18.29 Found: C 51.15; H 3.51; N 18.54.

1-[2-(4-fluorophenyl)-5-(4-methyl-1,2,3-thiadiazol-5-yl)-1,3,4-oxadiazol-3(2*H*)-yl]ethan-1-one (**21**)

White powder, Yield: 59%, M.p.: 94 °C; IR: 3060 (CH_arom_), 2970 (CH_aliph_), 1645 (C=O), 1510 (C=N), 1211, 1038 (C-OC); ^1^H NMR (600 MHz, DMSO-*d_6_*): 2.24 (s, 3H, CH_3_), 2.93 (s, 3H, CH_3_), 7.22 (s, 1H, CH_oxadiazole_), 7.29–7.32 (m, 2H, ArH), 7.59–7.62 (m, 2H, ArH); ^13^C NMR (150 MHz, DMSO-*d_6_*): 11.56 (CH_3_), 15.46 (CH_3_), 91.96 (CH_oxadiazole_), 116.37, 129.82, 130.48, 145.38 (6C_ar_), 155.07 (C_oxadiazole_), 158.78, 160.44 (2C_ar_), 163.81 (C=O); Anal. calc. for C_13_H_11_FN_4_O_2_S (306.32) (%): C 50.97; H 3.62; N 18.29. Found: C 50.25; H 3.70; N 18.59.

1-[2-(3-ethoxy-4-hydroxyphenyl)-5-(4-methyl-1,2,3-thiadiazol-5-yl)-1,3,4-oxadiazol-3(2*H*)-yl]ethan-1-one (**22**)

White powder, Yield: 64%, M.p.: 94 °C; IR: 3078 (CH_arom_), 2970 (CH_aliph_), 1636 (C=O), 1512 (C=N), 1213, 1042 (C-OC); ^1^H NMR (600 MHz, DMSO-*d_6_*): 1.28–1.30 (t, 3H, CH_3_, *J* = 6 Hz), 2.27 (s, 3H, CH_3_), 2.87 (s, 3H, CH_3_), 4.05–4.09 (q, 2H, CH_2_, *J* = 12 Hz, *J* = 6 Hz), 8.08–7.10 (m, 1H, ArH), 7.18 (s, 1H, CH_oxadiazole_), 7.26–7.27 (m, 1H, ArH), 7.53–7.59 (m, 1H, ArH), 9.97 (s, 1H, OH); ^13^C NMR (150 MHz, DMSO-*d_6_*): 14.88 (CH_3_), 15.00 (CH_3_), 20.78 (CH_3_), 64.68 (CH_2_), 92.30 (CH_oxadiazole_), 112.87, 119.40, 123.78, 134.56, 137.53, 149.72, 150.81 (7C_ar_), 155.24 (C_oxadiazole_), 158.76 (C_ar_), 163.34 (C=O); Anal. calc. for C_15_H_16_N_4_O_4_S (348.38) (%): C 51.71; H 4.63; N 16.08. Found: C 50.85; H 4.31; N 16.50.

1-[2-(2-bromo-6-hydroxyphenyl)-5-(4-methyl-1,2,3-thiadiazol-5-yl)-1,3,4-oxadiazol-3(2*H*)-yl]ethan-1-one (**23**)

White powder, Yield: 70%, M.p.: 110 °C; IR: 3074 (CH_arom_), 2939 (CH_aliph_), 1683 (C=O), 1598 (C=N), 1202, 1012 (C-OC); ^1^H NMR (600 MHz, DMSO-*d_6_*): 2.23 (s, 3H, CH_3_), 2.91 (s, 3H, CH_3_), 7.20 (s, 1H, CH_oxadiazole_), 7.24–7.26 (m, 1H, ArH), 7.74–7.76 (m, 1H, ArH), 7.86–7.91 (m, 1H, ArH), 10.03 (s, 1H, OH); ^13^C NMR (150 MHz, DMSO-*d_6_*): 14.53 (CH_3_), 23.16 (CH_3_), 96.46 (CH_oxadiazole_), 115.72, 115.95, 119.36, 123.60, 125.24, 133.28, 142.24, 148.40 (7C_ar_), 156.52 (C_oxadiazole_), 158.18 (C=O); Anal. calc. for C_13_H_11_BrN_4_O_3_S (383.22) (%): C 40.74; H 2.89; N 14.62. Found: C 40.25; H 3.10; N 14.50.

1-[2-(3-iodo-4-hydroxy-5-metoxyphenyl)-5-(4-methyl-1,2,3-thiadiazol-5-yl)-1,3,4-oxadiazol-3(2*H*)-yl]ethan-1-one (**24**)

White powder, Yield: 71%, M.p.: 120 °C; IR: 3078 (CH_arom_), 2970 (CH_aliph_), 1683 (C=O), 1592 (C=N), 1212, 1042 (C-OC); ^1^H NMR (600 MHz, DMSO-*d_6_*): 2.24 (s, 3H, CH_3_), 2.93 (s, 3H, CH_3_), 3.87 (s, 3H, OCH_3_), 7.15–7.17 (d, 1H, ArH, *J* = 12 Hz), 7.56–7.60 (m, 1H, ArH), 8.00 (CH_oxadiazole_), 9.93 (s, 1H, OH); ^13^C NMR (150 MHz, DMSO-*d_6_*): 15.01 (CH_3_), 20.94 (CH_3_), 56.92 (OCH_3_), 112.25 (CH_oxadiazole_), 128.40, 132.82, 135.50, 141.98, 145.38 (5C_ar_), 152.40 (C_oxadiazole_), 158.69, 160.21, 167.67 (3C_ar_), 168.89 (C=O); Anal. calc. for C_14_H_13_IN_4_O_4_S (460.25) (%): C 36.53; H 2.85; N 12.17. Found: C 37.27; H 3.11; N 12.54.

1-[5-(4-methyl-1,2,3-thiadiazol-5-yl)-2-(2-chloro-6-nitrophenyl)-1,3,4-oxadiazol-3(2*H*)-yl]ethan-1-one (**25**)

White powder, Yield: 66%, M.p.: 120 °C; IR: 3025 (CH_arom_), 2970 (CH_aliph_), 1677 (C=O), 1525 (C=N), 1211, 1059 (C-OC); ^1^H NMR (600 MHz, DMSO-*d_6_*): 2.32 (s, 3H, CH_3_), 2.93 (s, 3H, CH_3_), 7.48–7.50 (d, 1H, ArH, *J* = 12 Hz), 7.91–7.93 (m, 1H, ArH), 8.33–8.36 (m, 1H, ArH), 8.40 (s, 1H, CH_oxadiazole_), 8.40–8.41 (m, 1H, ArH); ^13^C NMR (75 MHz, DMSO-*d_6_*): 14.31 (CH_3_), 14.95 (CH_3_), 90.40 (CH_oxadiazole_), 125.62, 127.19, 132.57, 134.07, 139.40, 147.14, 155.62; (7C_ar_), 155.24 (C_oxadiazole_), 159.79 (C_ar_), 163.47 (C=O); Anal. calc. for C_13_H_10_ClN_5_O_4_S (367.77) (%): C 42.46; H 2.74; N 19.04. Found: C 42.25; H 2.31; N 19.50.

1-[2-(2,3-dimethoxyphenyl)-5-(4-methyl-1,2,3-thiadiazol-5-yl)-1,3,4-oxadiazol-3(2*H*)-yl]ethan-1-one (**26**)

White powder, Yield: 67%, M.p.: 110 °C; IR: 3018 (CH_arom_), 2970 (CH_aliph_), 1661 (C=O), 1575 (C=N), 1218, 1005 (C-OC); ^1^H NMR (600 MHz, DMSO-*d_6_*): 2.27 (s, 3H, CH_3_), 2.93 (s, 3H, CH_3_), 3.73 (s, 3H, OCH_3_), 3.83 (s, 3H, OCH_3_), 6.97–6.99 (m, 1H, ArH), 7.11–7.14 (t, 1H, ArH, *J* = 12 Hz, *J* = 6 Hz), 7.717–7.18 (m, 1H, ArH), 7.25 (s, 1H, CH_oxadiazole_); ^13^C NMR (75 MHz, DMSO-*d_6_*): 15.48 (CH_3_), 21.63 (CH_3_), 56.24 (OCH_3_), 56.36 (OCH_3_), 104.96 (CH_oxadiazole_), 115.36, 120.29, 124.86, 127.28, 135.81, 142.25, 148.91 (7C_ar_), 153.06 (C_oxadiazole_), 160.31 (C_ar_), 163.24 (C=O); Anal. calc. for C_15_H_16_N_4_O_4_S (348.38) (%): C 51.71; H 4.63; N 16.08. Found: C 49.95; H 4.51; N 16.40.

1-[2-(2,4-dimethoxyphenyl)-5-(4-methyl-1,2,3-thiadiazol-5-yl)-1,3,4-oxadiazol-3(2*H*)-yl]ethan-1-one (**27**)

White powder, Yield: 69%, M.p.: 110 °C; IR 3014 (CH_arom_), 2970 (CH_aliph_), 1721 (C=O), 1588 (C=N), 1280, 1031 (C-OC); ^1^H NMR (600 MHz, DMSO-*d_6_*): 2.09 (s, 3H, CH_3_), 2.98 (s, 3H, CH_3_), 3.85 (s, 3H, OCH_3_), 3.88 (s, 3H, OCH_3_), 6.68–6.69 (m, 1H, ArH), 6.76–6.78 (m, 1H, ArH), 7.91–7.92 (d, 1H, ArH, *J* = 6 Hz), 8.47 (s, 1H, CH_oxadiazole_); ^13^C NMR (75 MHz, DMSO-*d_6_*): 15.51 (CH_3_), 20.95 (CH_3_), 56.31 (OCH_3_), 56.40 (OCH_3_), 107.46 (CH_oxadiazole_), 114.61, 118.54, 122.10, 128.14, 130.17, 135.93, 142.11 (7C_ar_), 154.02 (C_oxadiazole_), 160.08 (C_ar_), 163.42 (C=O); Anal. calc. for C_15_H_16_N_4_O_4_S (348.38) (%): C 51.71; H 4.63; N 16.08. Found: C 50.25; H 4.71; N 16.35.

1-[2-(3,4-dimethoxyphenyl)-5-(4-methyl-1,2,3-thiadiazol-5-yl)-1,3,4-oxadiazol-3(2*H*)-yl]ethan-1-one (**28**)

White powder, Yield: 71%, M.p.: 130 °C; IR: 3031 (CH_arom_), 2970 (CH_aliph_), 1739 (C=O), 1578 (C=N), 1217, 1037 (C-OC); ^1^H NMR (600 MHz, DMSO-*d_6_*): 2.66 (s, 3H, CH_3_), 2.99 (s, 3H, CH_3_), 3.84 (s, 3H, OCH_3_), 3.90 (s, 3H, OCH_3_), 7.10–7.11 (d, 1H, ArH, *J* = 6 Hz), 7.34–7.35 (m, 1H, ArH), 7.43–7.44 (d, 1H, ArH, *J* = 6 Hz), 8.14 (s, 1H, CH_oxadiazole_); ^13^C NMR (150 MHz, DMSO-*d_6_*): 15.52 (CH_3_), 23.17 (CH_3_), 56.93 (OCH_3_), 56.12 (OCH_3_), 109.8 (CH_oxadiazole_), 109.80, 112.28, 122.62, 126.52, 135.56, 146.32, 149.59 (7C_ar_), 151.54 (C_oxadiazole_), 160.24 (C_ar_), 163.72 (C=O); Anal. calc. for C_15_H_16_N_4_O_4_S (348.38) (%): C 51.71; H 4.63; N 16.08. Found: C 50.05; H 4.82; N 16.80.

1-[5-(4-methyl-1,2,3-thiadiazol-5-yl)-2-(5-nitrofuran-2-yl)-1,3,4-oxadiazol-3(2*H*)-yl]ethan-1-one (**29**)

White powder, Yield: 70%, M.p.: 170 °C; IR: 3016 (CH_arom_), 2970 (CH_aliph_), 1739 (C=O), 1537 (C=N), 1229, 1032 (C-OC); ^1^H NMR (600 MHz, DMSO-*d_6_*): 2.25 (s, 3H, CH_3_), 2.88 (s, 3H, CH_3_), 7.32–7.33 (d, 1H, ArH, *J* = 6 Hz), 7.40 (s, 1H, CH_oxadiazole_), 7.74–7.75 (d, 1H, ArH, *J* = 6 Hz); ^13^C NMR (75 MHz, DMSO-*d_6_*): 11.55 (CH_3_), 14.95 (CH_3_), 85.01 (CH_oxadiazole_), 99.74, 115.59, 137.15, 149.83, 155.52 (5C_ar_), 158.60 (C_oxadiazole_), 163.56 (C=O); Anal. calc. for C_11_H_9_N_5_O_5_S (323.28) (%): C 40.87; H 2.81; N 21.66. Found: C 40.25; H 3.31; N 21.50.

1-[5-(4-methyl-1,2,3-thiadiazol-5-yl)-2-(1*H*-pyrrol-2-yl)-1,3,4-oxadiazol-3(2*H*)-yl]ethan-1-one (**30**)

White powder, Yield: 59%, M.p.: 150 °C; IR: 3016 (CH_arom_), 2970 (CH_aliph_), 1735 (C=O), 1555 (C=N), 1202, 1068 (C-OC); ^1^H NMR (600 MHz, DMSO-*d_6_*): 2.29 (s, 3H, CH_3_), 2.93 (s, 3H, CH_3_), 6.57–6.58 (m, 1H, ArH), 7.32–7.33 (m, 1H, ArH), 7.86–7.87 (m, 1H, ArH), 8.85 (s, 1H, CH_oxadiazole_), 9.18 (s, 1H, NH); ^13^C NMR (150 MHz, DMSO-*d_6_*): 15.49 (CH_3_), 21.50 (CH_3_), 113.12 (CH_oxadiazole_), 116.33, 126.13, 129.59, 135.88, 139.65 (5C_ar_), 160.26 (C_oxadiazole_), 163.62 (C_ar_), 172.53 (C=O); Anal. calc. for C_11_H_11_N_5_O_2_S (277.30) (%): C 47.64; H 4.00; N 25.26. Found: C 48.75; H 3.91; N 25.90.

### 3.2. Microbiology

The methodology for microbiological tests has been previously described by our research group [32,34] and is consistent with the standards of the European Committee on Antimicrobial Susceptibility Testing (EUCAST) and Clinical and Laboratory Standards Institute guidelines [35,36]. We used strains from the American Type Culture Collection (ATCC) as a panel of reference and clinical or saprophytic microbial strains. All compound stocks were prepared by dissolving them in DMSO. Tests were repeated in triplicate and representative results are shown.

### 3.3. Cytotoxicity Studies

Cytotoxicity studies were performed with the use of the normal cell line L929 (murine fibroblasts and neoplastic cells), HepG2 (human liver cancer) and, A549 (human lung cancer). All stock solutions of the test compounds were dissolved in DMSO. The methodology of testing was described by our team in the previous article [37].

### 3.4. Lipophilicity

Experimental lipophilicity of the synthesized compounds **16**–**22** and **24**–**30** (Table 6 and Table 7) was determined with the use of reversed-phase thin-layer chromatography on 10 × 20 cm RP18 F_254_ plates from Merck (Darmstadt, Germany). The methodology of the process was described in our earlier work [32].

## 4. Discussion

*S. aureus* is of great clinical importance, as it causes infections ranging from superficial skin symptoms to systemic sepsis. Initially, penicillin was the drug of choice for staphylococcal infections. However, the increased incidence of resistance to this antibiotic led to the introduction of methicillin—the first semisynthetic penicillin. Unfortunately, methicillin-resistant *S. aureus* (MRSA) was discovered shortly after, now belonging to multi-drug resistant organisms (MDRO) [38,39]. It is one of the most important serious opportunistic human pathogens involved in nosocomial infections. Resistance to methicillin primarily derives from acquisition of the mecA gene, which encodes a modified penicillin-binding protein (PBP2a) with low affinity for beta-lactams [40] The treatment of MRSA infection is greatly challenging since it has developed the resistance to almost all types of antibiotics, especially from the beta-lactam group, including penicillins, cephalosporins, monobactams and carbapenems. MRSA strains have also developed resistance to various other clinically used antibiotics such as fluoroquinolones, macrolides, aminoglycosides, daptomycin, and clindamycin creating a great threat to global healthcare [38,39]. Moreover, recent studies indicates that MRSA is continuously evolving as a superbug. Therefore, the development of new therapies for eradication of this microorganism is of great importance. Due to this high activity of compound **29** against *S. aureus*, ATCC 43300 is significant.

Additionally, on the basis of our microbiological tests, both these presented in this article and those already published [32], it can be concluded that acylhydrazones, compared to 3-acetyl-1,3,4-oxadizolines, show greater activity against Gram-positive and Gram-negative bacterial strains, but lower activity against fungi.

A similar situation was observed in the article by Zorzi et al. [41], where the cyclization reaction was performed with [*N*-(5-nitrofuran-2-yl)methylene]benzhydrazide, which resulted in the synthesis of a series of 3-acetyl-5-(substitutedphenyl)-2-(5-nitrofuran-2-yl)-2,3-dihydro-1,3,4-oxadiazoles, which showed lower antimicrobial activity in comparison with acylhydrazones. Especially, this dependence was observed for the compound with the *t*-butyl substituent where the hydrazone against *S. aureus* ATCC 29213 was active (MIC = 4–8 µM) and the corresponding 1,3,4-oxadiazole showed no activity [41], In two other studies, the cyclization of acylhydrazones into corresponding 3-acetyl-1,3,4-oxadiazolines resulted in lower values of bioactivity against *S. aureus* strain [12,16]

The bactericidal effect against Gram-positive bacteria for acylhydrazone with a 5-nitrofuroyl moiety was in the range of MIC from 3.91 to 62.5 µg/mL [32], whereas for the corresponding 1,3,4-oxadiazole, it was 3.91–250 µg/mL. The synthesized group of 1,3,4-oxadiazole derivatives showed higher activity only against fungi. This fact can be observed in case of compounds **8** and **23**. Compound **23** showed the MIC values in the range of 125–500 µg/mL, and the corresponding acylhydrazone did not have this activity at all. The most active in both groups were compounds with a 5-nitrofuroyl moiety, which can be found in well-known medicines such as nitrofurantoin or furazolidone.

These results confirm the relationship presented in our previous two articles [34,42]. The fact that the 5-nitrofuran-2-yl moiety in the second position of the 1,3,4-oxadiazoline ring determines the activity of these derivatives and the cyclization of the *N*-heteroarylidene analogues to their 3-acetyl-1,3-4-oxadiazoline analogues resulted in a dramatic decrease of antibacterial activity [34,42]. On the basis of the conducted research, it can therefore be concluded that acylhydrazones are more active in terms of antimicrobial activity than the corresponding 1,3,4-oxadiazole derivatives.

Nevertheless, it seems justified to carry out additional tests, due to which it will be possible to establish the relationship between the activity and chemical structure of 3-acetyl-1,3,4-oxadiazolines.

## 5. Conclusions

In summary, the simple cyclization reaction of the corresponding acylhydrazones, described earlier by our research group [32], in the acetic anhydride, allowed to obtain a series of new 3-acetyl-2,5-disubstituted-1,3,4-oxadiazoline derivatives. This reaction was carried out with the efficiency of about 60%, which can be considered as satisfactory result. Among this group, one compound, namely **29** with 5-nitrofuran-2-yl moiety, deserves special attention. It showed the highest activity, especially against strains of *Staphylococcus* spp., including multi-drug resistant microorganisms belonging to methicillin-resistant *S. aureus*. Moreover, cytotoxicity tests showed that this compound showed low cytotoxicity. Conversely, the conducted lipophilicity studies showed that the presence of a halogen atom in the structure significantly influences the lipophilicity of the compound. We believe that the combination of this information provides a good foundation for the synthesis of new groups of compounds with potential antimicrobial activity.

## Figures and Tables

**Table 1:** The activity data of compounds **16**–**30** expressed as MIC (MBC or MFC) (µg/mL) and MBC/MIC or MFC/MIC values against the reference strains of microorganisms.

Species/ Compound No		MIC (MBC or MFC) (µg/mL) and MBC/MIC or MFC/MIC Compounds and Reference Substances										
		19	21	22	23	24	25	29	CIP/ NY *	NIT	CFX	APC
**Gram-Positive Bacteria**	*Staphylococcus* *aureus* ATCC 25923	1000 (>2000) {>2}	-	-	1000 (>2000) {>2}	-	-	15.62 (31.25) {**2**}	0.48 (0.48)	15.62 (15.62)	0.49	nd
	*Staphylococcus* *aureus* ATCC 6538	-	-	-	500 (>2000) {>4}	-	125 (>2000) {>8}	15.62 (31.25) {**2**}	0.24 (0.24)	15.62 (15.62)	0.98	nd
	*Staphylococcus* *aureus* ATCC 43300	1000 (>2000) {>2}	-	-	1000 (>2000) {>2}	-	-	15.62 (31.25) {**2**}	0.24 (0.24)	7.81 (15.62)	nd	nd
	*Staphylococcus* *aureus* ATCC 29213	-	-	-	500 (>2000) {>4}	-	500 (>2000) {>4}	15.62 (15.62) {**1**}	0.48 (0.48)	nd	nd	nd
	*Staphylococcus* *epidermidis* ATCC 12228	1000 (>2000) {>2}	1000 (>2000) {>2}	1000 (>2000) {>2}	500 (2000) {**4**}	250 (1000) {8}	62.5 (250) {**4**}	3.91 (15.62) {**4**}	0.12 (0.12)	3.91 (7.81)	0.24	nd
	*Enterococcus* *faecalis* ATCC 29212	1000 (>2000) {>2}	1000 (>2000) {>2}	-	1000 (>2000) {>2}	-	250 (>2000) {>4}	250 (500) {**2**}	0.98 (1.95)	nd	nd	nd
	*Micrococcus* *luteus* ATCC 10240	1000 (>2000) {>2}	1000 (>2000) {>2}	-	125 (2000) {8}	-	-	250 (1000) {**4**}	0.98 (1.95)	62.5 (62.5)	0.98	nd
	*Bacillus* *subtilis* ATCC 6633	1000 (>2000) {>2}	-	1000 (>2000) {>2}	1000 (>2000) {>2}	-	-	15.62 (15.62) {**1**}	0.03 (0.03)	3.91 (3.91)	15.62	62.5
	*Bacillus* *cereus* ATCC 10876	1000 (>2000) {>2}	-	1000 (>2000) {>2}	250 (>2000) {>8}	500 (>2000) {>4}	-	31.25 (62.5) {**2**}	0.06 (0.12)	7.81 (15.62)	31.25	nd
**Gram-Negative Bacteria**	*Bordetella* *bronchiseptica* ATCC 4617	-	-	-	62.5 (1000) {16}	-	-	500 (2000) {**4**}	0.98 (0.98)	125 (>1000)	nd	nd
	*Klebsiella* *pneumoniae* ATCC 13883	-	-	-	1000 (>2000) {>1}	-	-	250 (>2000) {>8}	0.12 (0.24)	15.62 (31.25)	nd	nd
	*Proteus* *mirabilis* ATCC 12453	-	-	-	500 (>2000) {>2}	-	-	500 (>2000) {>4}	0.03 (0.03)	62.5 (125)	nd	nd
	*Salmonella* *typhimurium* ATCC 14028	-	-	-	1000 (>2000) {>2}	-	-	125 (250) {**2**}	0.06 (0.06)	31.25 (62.5)	nd	nd
	*Escherichia* *coli* ATCC 25922	-	-	-	1000 (>2000) {>2}	-	-	62.5 (125) {**2**}	0.004 (0.008)	7.81 (15.62)	nd	nd
**Fungi**	*Candida* *albicans* ATCC 2091	-	-	-	125 (125) {**1**}	500 (>2000) {>4}	-	62.5 (62.5) {**1**}	0.24 * (0.48)	na	na	na
	*Candida* *albicans* ATCC 10231	-	-	-	125 (125) {**1**}	500 (1000) {2}	-	125 (500) {**4**}	0.48 * (0.48)	na	na	na
	*Candida* *parapsilosis* ATCC 22019	-	-	-	250 (1000) {**4**}	500 (>2000) {>4}	1000 (>2000) {>2}	500 (1000) {**2**}	0.24 * (0.48)	na	na	na
	*Candida* *glabrata* ATCC 90030	-	-	-	500 (500) {**1**}	1000 (>2000) {>2}	-	1000 (>2000) {>2}	0.24 * (0.48)	na	na	na
	*Candida* *krusei* ATCC 14253	-	-	-	500 (500) {**1**}	1000 (>2000) {>2}	-	500 (1000) {**2**}	0.24 * (0.24)	na	na	na

‘-’ no activity; nd, not determined; na, not applicable. The standard chemotherapeutics used as positive controls: ciprofloxacin (CIP), nitrofurantoin (NIT), cefuroxime (CFX) and ampicillin (APC) for bacteria and nystatin (NY *) for fungi. Compounds with bactericidal effect (MBC/MIC ≤ 4) or fungicidal effect (MFC/MIC ≤ 4) are marked with bold font. No bioactivity—MIC > 1000 µg/mL; mild bioactivity—MIC = 501–1000 µg/mL; moderate bioactivity—MIC = 126–500 µg/mL; good bioactivity—MIC = 26–125 µg/mL; strong bioactivity—MIC = 10–25 µg/mL; very strong bioactivity—MIC < 10 µg/mL; * nystatin activity.

**Table 2:** The cell proliferation in % according to control after 24 and 48 h exposition on studied compounds in L929 cell line.

Dose/Compound	24		25		29	
	After 24 h	After 48 h	After 24 h	After 48 h	After 24 h	After 48 h
**200 µM**	75%	83%	69%	59%	77%	84%
**150 µM**	101%	97%	71%	67%	75%	79%
**100 µM**	84%	90%	57%	72%	62%	71%
**50 µM**	81%	74%	63%	61%	94%	102%
**25 µM**	84%	79%	60%	69%	68%	69%
**12 µM**	107%	96%	79%	84%	61%	78%
**6 µM**	110%	103%	87%	92%	99%	82%

Green color—increase in the metabolic activity of the cell; red color—decrease in the metabolic activity of the cell.

**Table 3:** The cell proliferation in % according to control after 24 and 48 h exposition on studied compounds in A549 cell line.

Dose/Compound	24		25		29	
	After 24 h	After 48 h	After 24 h	After 48 h	After 24 h	After 48 h
**200 µM**	77%	68%	89%	73%	86%	97%
**150 µM**	89%	104%	102%	75%	112%	131%
**100 µM**	92%	92%	88%	79%	114%	91%
**50 µM**	74%	67%	96%	97%	93%	127%
**25 µM**	97%	103%	91%	118%	117%	125%
**12 µM**	109%	121%	78%	141%	128%	147%
**6 µM**	87%	98%	83%	120%	116%	131%

Green color—increase in the metabolic activity of the cell.

**Table 4:** The cell proliferation in % according to control after 24 and 48 h exposition on studied compounds in HepG2 cell line.

Dose/Compound	24		25		29	
	After 24 h	After 48 h	After 24 h	After 48 h	After 24 h	After 48 h
**200 µM**	88%	97%	80%	99%	79%	96%
**150 µM**	97%	105%	84%	118%	85%	91%
**100 µM**	102%	98%	76%	97%	88%	131%
**50 µM**	85%	107%	81%	104%	96%	97%
**25 µM**	96%	108%	88%	103%	92%	141%
**12 µM**	105%	116%	93%	111%	91%	129%
**6 µM**	113%	126%	99%	119%	107%	112%

Green color—increase in the metabolic activity of the cell.

**Table 5:** The log P values from the literature [31] and the calculated R_M0_ values for the reference substances.

	Log P	R_M0_	*S*	*r* ^2^	Φ
**Acetone-Water**					
Acetaminophen	0.46	0.73	−0.02	0.9787	39.78
Salicylamide	1.28	1.32	−0.02	0.9853	55.63
Nitrophenol	1.91	1.99	−0.03	0.9800	63.72
Ethyl hydroxybenzoate	2.47	1.96	−0.03	0.9816	64.90
Thymol	3.30	3.25	−0.04	0.9910	73.20
Phenyl salicylate	3.80	4.42	−0.06	0.9779	75.39
**Acetonitrile-Water**					
Acetaminophen	0.46	1.09	−0.03	0.9138	39.00
Salicylamide	1.28	1.15	−0.03	0.9922	43.38
Nitrophenol	1.91	1.79	−0.04	0.9937	50.80
Ethyl hydroxybenzoate	2.47	1.81	−0.03	0.9889	53.75
Thymol	3.30	2.27	−0.03	0.9929	68.02
Phenyl salicylate	3.80	2.41	−0.03	0.9488	74.91
**1,4-Dioxane-Water**					
Acetaminophen	0.46	0.17	−0.01	0.9942	14.56
Salicylamide	1.28	0.61	−0.02	0.9706	39.36
Nitrophenol	1.91	1.18	−0.02	0.9942	51.84
Ethyl hydroxybenzoate	2.47	1.61	−0.03	0.9818	56.43
Thymol	3.30	2.99	−0.04	0.9854	70.66
Phenyl salicylate	3.80	3.45	−0.05	0.9954	76.67
**Methanol-Water**					
Acetaminophen	0.46	0.72	−0.02	0.9540	37.89
Salicylamide	1.28	1.04	−0.02	0.9817	54.27
Nitrophenol	1.91	1.50	−0.02	0.9856	64.57
Ethyl hydroxybenzoate	2.47	2.11	−0.03	0.9906	70.27
Thymol	3.30	3.14	−0.04	0.9857	82.20
Phenyl salicylate	3.80	4.09	−0.05	0.9782	88.57

Φ is the amount of organic modifier in the mobile phase; R_M0_ and *S* are the intercept and slope of the linear regression equation; *r*^2^ is the correlation coefficient.

**Table 6:** The R_M0_ values of the synthesized 1,3,4-oxadiazoline derivatives.

Compound No.	R_M0_	*S*	*r* ^2^	Φ
**Acetone-Water**				
16	1.84	−0.03	0.9904	66.04
17	3.35	−0.05	0.9833	71.02
18	3.58	−0.05	0.9929	72.76
19	2.96	−0.04	0.9898	69.07
20	3.16	−0.05	0.9916	70.13
21	2.92	−0.04	0.9958	70.58
22	3.19	−0.05	0.9856	67.06
24	3.86	−0.06	0.9772	65.71
25	2.55	−0.04	0.9835	66.65
26	1.62	−0.03	0.9792	69.40
27	2.83	−0.04	0.9667	76.18
28	0.68	−0.01	0.9661	48.86
29	2.86	−0.04	0.9723	66.51
30	1.84	−0.03	0.9921	62.72
**Acetonitrile-Water**				
16	1.27	−0.02	0.9231	57.39
17	2.26	−0.03	0.9790	72.90
18	2.74	−0.04	0.9809	74.16
19	2.22	−0.03	0.9694	68.52
20	2.22	−0.03	0.9648	70.76
21	2.29	−0.03	0.9769	69.33
22	2.54	−0.04	0.9181	65.80
24	2.20	−0.04	0.9641	61.17
25	2.08	−0.04	0.9840	59.15
26	1.74	−0.03	0.9201	59.10
27	3.04	−0.04	0.9627	72.33
28	2.90	−0.05	0.9258	61.62
29	2.60	−0.04	0.9899	59.18
30	1.37	−0.03	0.9916	51.82
**1,4-Dioxane-Water**				
16	1.69	−0.03	0.9715	56.78
17	2.95	−0.05	0.9955	65.35
18	3.15	−0.04	0.9886	70.54
19	2.59	−0.04	0.9609	65.79
20	2.90	−0.03	0.9919	67.85
21	2.67	−0.04	0.9868	68.11
22	2.61	−0.04	0.9903	62.34
24	2.54	−0.04	0.9967	60.19
25	2.14	−0.04	0.9849	58.74
26	1.30	−0.02	0.9980	56.70
27	3.29	−0.05	0.9980	70.21
28	2.68	−0.04	0.9684	62.23
29	1.56	−0.03	0.9830	54.86
30	1.07	−0.02	0.9873	50.57
**Methanol-Water**				
16	2.59	−0.04	0.9823	73.28
17	3.69	−0.04	0.9782	87.44
18	3.86	−0.05	0.9863	85.82
19	3.06	−0.04	0.9617	82.65
20	3.38	−0.05	0.9905	82.39
21	3.19	−0.04	0.9844	81.85
22	3.38	−0.04	0.9905	82.39
24	4.46	−0.05	0.9896	83.25
25	3.52	−0.05	0.9469	75.45
26	2.31	−0.03	0.9809	72.70
27	2.01	−0.03	0.9771	71.79
28	1.22	−0.02	0.9608	59.80
29	2.67	−0.04	0.9950	75.85
30	1.96	−0.03	0.9851	70.94

Φ is the amount of organic modifier in the mobile phase; R_M0_ and *S* are the intercept and slope of the linear calibration equation; *r*^2^ is the correlation coefficient.

**Table 7:** The log P_EXP_ values of the synthesized 1,3,4-oxadiazoline derivatives.

Compound No.	Log P_acetone_	Log P_acetonitrile_	Log P_1,4-dioxane_	Log P_methanol_
**16**	1.81	1.14	2.22	2.66
**17**	3.16	3.32	3.41	3.69
**18**	3.37	4.40	3.59	3.85
**19**	2.81	3.24	3.07	3.10
**20**	2.99	3.24	3.36	3.40
**21**	2.78	3.39	3.14	3.22
**22**	3.02	3.94	3.08	3.40
**24**	3.62	3.20	3.02	4.41
**25**	2.44	2.93	2.64	3.53
**26**	1.62	2.11	1.86	2.40
**27**	2.70	5.05	3.72	2.12
**28**	0.78	4.73	3.15	1.38
**29**	2.72	4.09	2.10	2.74
**30**	1.81	1.34	1.64	2.07

## Data Availability

Data is contained within the article or Supplementary Materials.

## Supplementary Materials

The following are available online at https://www.mdpi.com/article/10.3390/ijms222413669/s1.
