# Supplementary material for: Biological Activity, Lipophilicity and Cytotoxicity of Novel 3-Acetyl-2,5-disubstituted-1,3,4-oxadiazolines
Source: Int J Mol Sci. 2021 Dec 20;22(24):13669. doi: 10.3390/ijms222413669 (PMC8704594; doi:10.3390/ijms222413669)
Supplement: Supplementary file 1 [file ijms-22-13669-s001.zip › ijms-1476092-supplementary.pdf]

International Journal of
Molecular Sciences

MDPI

Supplementary Materials

Biological Activity, Lipophilicity and Cytotoxicity of Novel 3-Acetyl-2,5-disubstituted-1,3,4-oxadiazolines

Kinga Paruch [1,*], Anna Biernasiuk [2], Anna Berecka-Rycerz [3], Anna Hordyjewska [4] and Łukasz Popiołek [1]

1 Chair and Department of Organic Chemistry, Faculty of Pharmacy, Medical University of Lublin, 4A Chodźki Street, 20-093 Lublin, Poland; lukasz.popiolek@umlub.pl
2 Chair and Department of Pharmaceutical Microbiology, Faculty of Pharmacy, Medical University of Lublin, 1 Chodźki Street, 20-093 Lublin, Poland; anna.biernasiuk@umlub.pl
3 Chair and Department of Medicinal Chemistry, Faculty of Pharmacy, Medical University of Lublin, 4 Jaczewskiego Street, 20-093 Lublin, Poland; anna.berecka@umlub.pl
4 Chair and Department of Medicinal Chemistry, Faculty of Medical Sciences, Medical University of Lublin, 4A Chodźki Street, 20-093 Lublin, Poland; anna.hordyjewska@umlub.pl
* Correspondence: kinga.paruch@umlub.pl

Microbiology - In vitro **Antimicrobial Assay Results**

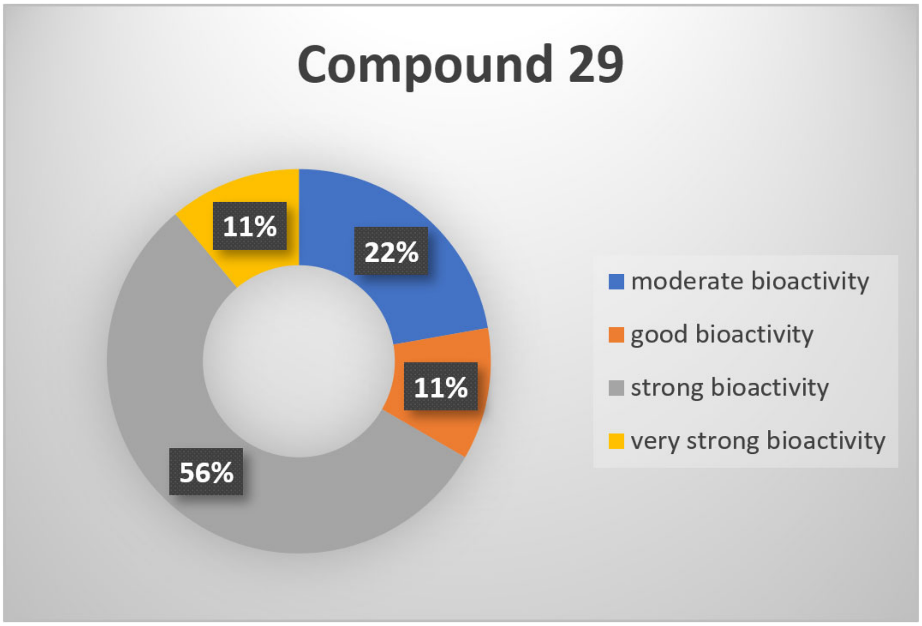

(**a**)

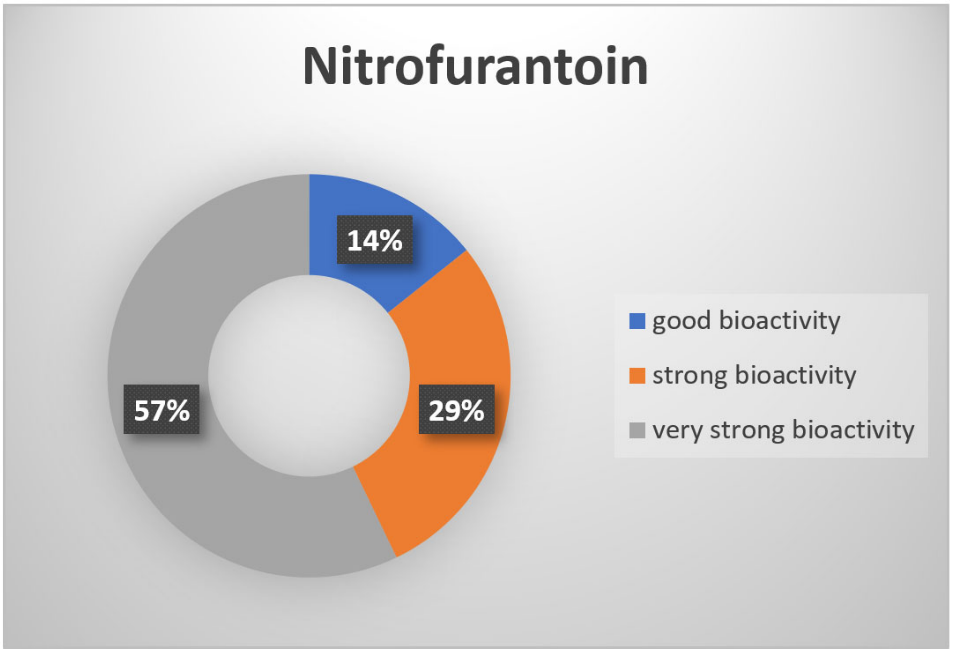

(**b**)

Figure S1. The antimicrobial activity data of the: (**a**) compound **29** and (**b**) nitrofurantoin, against tested reference Gram-positive bacterial strains. (no bioactivity – MIC >1000 µg/mL; mild bioactivity – MIC = 501–1000 µg/mL; moderate bioactivity – MIC = 126–500 µg/mL; good bioactivity – MIC = 26–125 µg/mL; strong bioactivity – MIC = 10–25 µg/mL; very strong bioactivity – MIC <10 µg/mL).

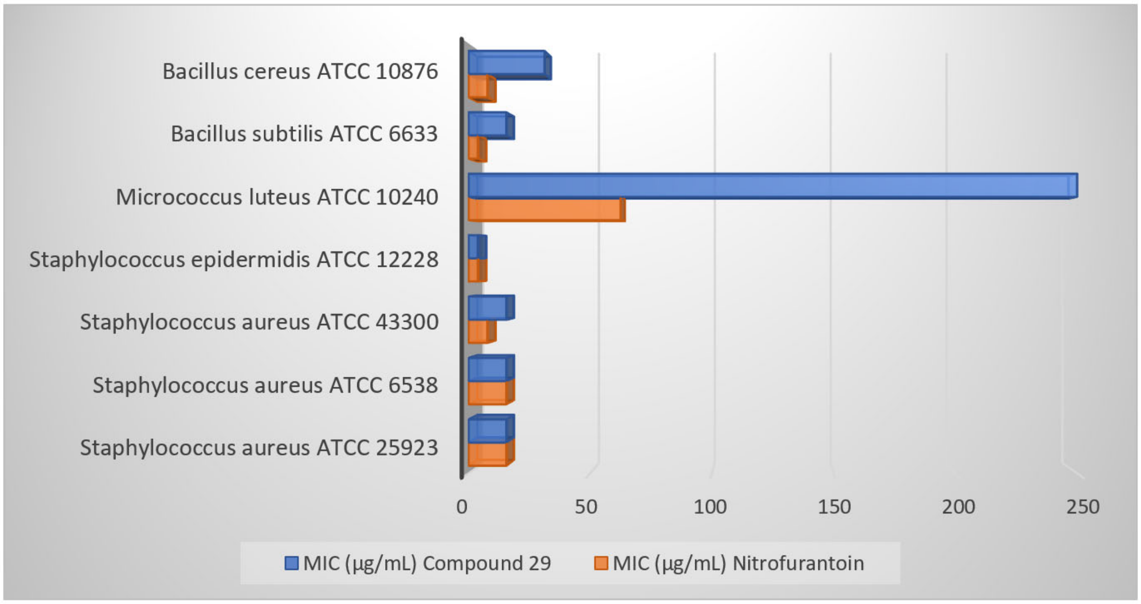

Figure S2. The antimicrobial activity assay results (MIC, µg/mL) for the compound **29** in comparison with nitrofurantoin against the reference Gram-positive bacterial strains.
